# Can reproductive health services be used to screen for sexual and gender-based violence in post-conflict Northern Uganda? – a pilot study

**DOI:** 10.4314/ahs.v24i1.13

**Published:** 2024-03

**Authors:** Keneth Opiro, Francis Pebolo Pebalo, Neil J Scolding, Charlotte Scolding

**Affiliations:** 1 Faculty of Medicine, Gulu University, Uganda; 2 University of Bristol, UK

**Keywords:** SGBV, resource-poor setting, Screening

## Abstract

**Background:**

Sexual and gender-based violence (SGBV), including rape and child sexual abuse, remains a significant challenge in post-conflict northern Uganda. Many victims have never sought help. Consequently, the scale of the problem is not known, and SGBV victims' injuries, both psychological and physical, remain hidden and unresolved.

**Objectives:**

We aimed to explore whether health workers in rural Reproductive Health Services (RHS), following specific training, could provide a valuable resource for SGBV screening and subsequent referral to targeted services.

**Methods:**

Our project had three elements. First, RHS workers were trained to use a questionnaire to screen subjects for past SGBV Second, the screening questionnaire was used by RHS workers over a 3-month period, and the data collected were analysed to explore whether the screening approach was an effective one in this setting, and to record the scale and nature of the problem. Third, victims detected were offered referral as appropriate to hospital services or to a dedicated SGBV ActionAid shelter.

**Results:**

Of 1656 women screened, 778 (47%) had suffered SGBV: 123 rape, and 505 non-sexual violence. 1,254 (76%) had been directly or indirectly affected by conflict experiences; 1066 had lived in internally displaced persons camps. 145 (9%) requested referral to Gulu SGBV Shelter; 25 attended the shelter and received assistance, and 20 others received telephone counselling.

**Conclusion:**

Undetected SGBV remains a significant problem in post-conflict northern Uganda. RHS workers, following specific training, can effectively screen for and identify otherwise unrecognised survivors of SGBV. This matters because without ongoing detection, survivors have no opportunity for resolution, healing or help.

## Introduction

The civil war in Northern Uganda started in the early 1980s and lasted over twenty years. Rebels in the North continued to pursue a campaign of terror after peace had been established in the rest of Uganda, only resolving from 2007-8 onwards. Sexual and gender-based violence (SGBV), including rape and child sexual abuse, was prominent and persisted in many IDP camps in the region, and has continued into the post-conflict period 1-5. It remains a significant challenge in the whole of the region including but not exclusively within refugee settlements. SGBV in IDP camps has been studied 6, 7 but there is very little information about survivors of these abuses in non-refugee settings - in particular, the scale of the problem. Regional cultural practices and a lack of awareness of women's rights underway relating to SGBV can lead to abuses being seen as “normalised” in a cultural context 2, 4, 6, 8-15.

Assessing the scale of SGBV must underpin efforts to address the problem, but is extremely challenging. Assessments of SGBV prevalence derived either from police reports or from health systems data underestimate the true scale by between 11- and 128-fold 16. The reasons for this are complex and multiple, perhaps particularly in a LMIC setting 17, but not the least important is that the nature of the experiences can cause personal shame and stigma, as well as fear of financial loss and of retribution by the perpetrator, often to the extent that many of the victims do not seek help from health-related services, including psychological or mental health services; and their injuries, both psychological and physical, remain undetected and unaddressed - as do issues of pregnancy and children resulting from SGBV experiences 5, 13, 16.

It is generally accepted that methods of screening populations to identify victims would therefore be valuable 7 16-18. However, while various screening approaches have been proposed and validated in resource-rich settings, they cannot be applied in LMIC settings, which present particular challenges 16, 18. Screening programmes directed explicitly and exclusively towards SGBV might appear the ideal approach, but are problematic: in one study in a LMIC refugee setting, while almost 90% females consented to take part, only 15% could in practice participate, for various reasons, particularly lack of private space in the clinics 18.

Most women do attend Reproductive Health Service clinics during their adolescent or adult years, and such clinics by their nature commonly have private space. These clinics could therefore give health workers an opportunity to screen for and manage SGBV-related problems. However, many health workers are not fully trained in the detection and management of SGBV, which reduces their ability to care for this group.

We hypothesized that health workers in Reproductive Health Service (RHS) clinics could represent a valuable resource for screening their patient cohort for SGBV, such that a significant number of women and girls could be actively detected and offered treatments (above and beyond those who had already self-presented). This would first, however, require some specific training in SGBV.

We provided a training in the form of workshops so that health workers in RHS were then able to use a bespoke questionnaire in the setting of the existing health systems in which they work, to conduct such a screening.

The current pilot study had three elements. First, health workers in Reproductive Health Services in Gulu Regional Referral Hospital and in Awach Health Centre IV (Health Sub District) in Northern Uganda were trained in the knowledge and skills needed to screen for and identify women who had experienced SGBV using a questionnaire-based approach.

Second, the screening questionnaire was used by health workers over a 3-month period, and the emerging data were used to explore the scale and nature of the problem. Third, those detected were offered referrals to hospital services where appropriate, and/or to existing SGBV support services, namely those provided by the Action-Aid SURGE (Strengthening Uganda's Response to Gender Equality) programme at the SGBV shelter in Gulu. The HWs also contributed to awareness of SGBV as a recognized and common problem for women and gave them a chance to talk about experiences confidentially.

## Methods

### Setting

Our pilot study was designed and delivered in partnership with Gulu University and Gulu Regional Referral Hospital in northern Uganda. The Reproductive Health Service teams who were to undertake SGBV screening were those from two centres, Gulu Regional Referral Hospital and from the nearby Awach Health Centre IV, the two main government health facilities in Gulu District. These two teams provide Reproductive Health Service clinics for a population of approximately 1.5 million.

### Training

A total of twenty nine health workers in Reproductive Health Services attended the training workshops, in two groups of 15 and 14 health workers, from Gulu Regional Referral Hospital and from Awach Health Centre IV respectively. They were trained through one-day workshops held on consecutive days. Their background and previous training were varied – some were midwives, some nurses and others were clinical officers. A small number had already received SGBV training.

The workshops were based on material from a UNHCR training package on SGBV 19, with added elements related to forensic examinations, police documentation and legal aspects, counselling and support, in a Ugandan context.

The training was delivered by two Gulu University lecturers in Obstetrics and Gynaecology, two visiting professors at Gulu University from the UK, a Kampala-based senior consultant forensic pathologist with the Uganda Ministry of Health, and two members of staff from the SURGE SGBV shelter – a social worker (with specific psychology and counselling training in SGBV) and a lawyer (also specialising in the field of SGBV and women's rights). Basic education on SGBV rights under the law, how to complete a police report form, forensic specimen collection, and basic counselling of victims were included in the training.

A main focus of the training workshops was familiarity with the questionnaire, consent, and use of a confidentiality algorithm.

### Questionnaires

A questionnaire was designed based on that devised and used in Rwamwanja Refugee Settlement, Kamwenge District, western Uganda by the Population Council (PC) in 2016 7. We added questions relating to a variety of different SGBV experiences, and questions about experiences of conflict, as this is a post-conflict population and there is evidence that incidence of SGBV rises during and especially soon after conflict in affected populations 1, 4, 14, 20, 21.

In addition, we included a consent form adapted from that designed by the ICRC (International Committee of the Red Cross).

An algorithm designed specifically for the purpose by UNFPA and WAVE was used to check that there was sufficient privacy for confidentiality to be provided during the patient encounter, a fundamental need for interviews of this nature. Privacy was further ensured by conducting one-to-one screening in a room where no third party was allowed except with consent of the person being screened. Health workers were specifically trained in maintaining privacy and confidentiality especially, given the sensitive nature of the topic.

Health workers were also trained in simple counselling and, where necessary, a referral pathway for those who found the interviews distressing or responded positively to the opportunity for further counselling by specialists in the field.

At the beginning of the Reproductive Health Clinic, the health workers were encouraged to present a general talk on SGBV to patients waiting; the women screened were those who consented for the SGBV interviews after this talk. Questionnaire data collected during the project implementation provided a pragmatic estimate of the current prevalence of SGBV within a cross section of those women attending reproductive and gynaecological health services in the study regions, during a 3-month period. No particular age group or demographic was specified or excluded. No time limitation for how long ago the experiences had happened was specified.

Women who were attending for other reasons, between the ages of 15-80, but were found to have suffered SGBV were referred for appropriate care to hospital services (these were not specified in our questionnaire but included treatment for sexually transmitted diseases and ante- and post-natal care, as well as other gynaecological care related to their experiences). They were also referred, if they wished, for more specialised counselling, support for police and court processes, community follow-up, couple mediation or in a few cases, protection, as dictated by their needs.

Each of the health workers was given a supply of questionnaires for completion over a three-month period, during which the questionnaires were completed and consequent referrals made.

### Outcomes

The main outcome measure was number of women attending reproductive health services, who were found to screen positive for experience of SGBV.

Secondary outcomes were numbers of women who were referred to non-hospital-based facilities and received assistance from those services. The non-hospital based SGBV services to which referrals had been made were consulted, in order to determine the numbers of referred women who attended their referrals (always made on the basis of the patient's wishes).

### Ethics

The project proposal was submitted to Gulu University Research Ethics Committee (GUREC) and was approved [GUREC-002-19].

### Funding

The project was funded in part by the UK Foreign Office.

### Patient and Public involvement

Patients and the public were not involved in the design of this study.

A presentation was made after the completion of the project to health workers, local public health and regional health officials, so as to disseminate information and interest amongst stakeholders and decision makers.

## Results

In the three-month period of the study, and in the two health units, individual questionnaires were used to screen 1656 women. The age range of the respondents is shown in the figure4. Reproductive healthcare workers reported that the questionnaires were straightforward to use, and they made no major suggestions for changes.

### Primary outcomes

Analysis of the 1656 questionnaires revealed that 778 screened women (47%) had a history of SGBV

### Secondary outcomes

Of the 778 victims, 145 (9%) were referred at their request to Gulu SGBV Shelter under SURGE. Twenty-five (17%) of those referred attended the shelter and received assistance, and a further twenty (14%) received counselling by phone. Young mothers who had been victims of defilement received help (mainly financial and logistical) with the medical needs of their children, as well as with transport to hospital and/or other services.

### Other outcomes

#### Demographics

The largest age group screened were women between 20 and 30 years of age – 45% (748), (see below).

Of those screened, 68% (1,129) resided in rural areas.

**Figure:**
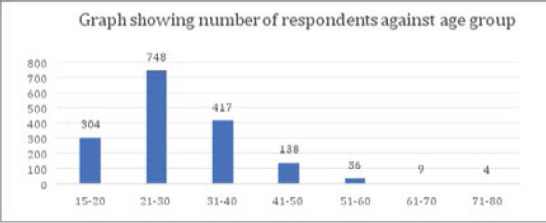


#### Types of SGBV experience

The questionnaire results revealed that victims had been subject to various types of SGBV. See Table 1, below.

**Table 1:** Numbers of victims (and percentages of the 778 SGBV sufferers) of various categories of SGBV. Each of these categories was non-exclusive: women may have answered in the affirmative in several categories

N^o^. victims	Percentage	SGBV experience
123	15.8%	Rape
126	16.2%	Forced intimacy other than rape
266	34.2%	Forced sex within an intimate relationship
82	10.5%	Forced marriage
322	41.3%	Sexual harassment
505	64.9%	Gender-based (non-sexual) violence

The total included several cases of defilement (defined in Ugandan law as forced sex with a child under the age of 18).

### Conflict experience

A large majority – 76% (1,254) – had been directly or indirectly affected by the insurgency (or other conflict experiences).

Of the cohort screened, 1066 (64%) had lived in IDP camps, and 170 (10%) had been abducted and experienced forced sex.

## Discussion

WHO 2013 22: “One key aspect [of exposure to violence and health consequences] is to identify opportunities to provide support and link women with other services they need – for example, when women seek sexual and reproductive health services (e.g. antenatal care, family planning, post-abortion care) or HIV testing, mental health and emergency services.”

We have shown that it is possible with a relatively simple approach (our questionnaire, and one day of additional training) to utilise existing women's health services effectively to discover cases of SGBV which had remained unreported and unassisted. The specifically trained health workers who administered the questionnaires to women attending reproductive health clinics were enthusiastic and well-motivated to deliver the SGBV screening. It was clear that it would be entirely feasible for screening and referral for SGBV cases to continue in terms of commitment of health workers, and therefore likely that significant ‘scaling up’ of this pilot project would be plausible. This confirms the findings of the Rwamwanja Settlement screening study in emergency settings in Western Uganda by the Population Council Group in 2015-2016, though the populations differ in location and in that these are not emergency settings 7.

Some 47% of those women screened had suffered SGBV of some sort, a higher-than-expected percentage exceeding global prevalence estimates from previous studies (approximately 36%) 22, 23. This level of ‘unknown’ SGBV experience in the community fits in well with global studies indicating that as few as 7% of SGBV victims reported their experience to a formal source 15, 16. (A very recent study in south-eastern Uganda suggested a figure of just under 40% 24). It also accords with studies in emergency settings in Uganda showing that survivors are willing to respond well to screening approaches and to disclose SGBV 17. Our study strongly suggested that there is a need for active continued screening, especially in consideration of those women who chose to be referred for help, who were previously not known to be victims, and who may well not otherwise have sought further help. Whilst screening appeared effective for giving women a new opportunity to gain assistance, a significant number of referred individuals did not contact the services to which they were referred. 148 women were referred (at their request), but only 45 of these made contact with the SURGE centre, either in person or by telephone.

No data were collected regarding more detailed demographics or contact information to enable follow-up enquiries, and so the reasons for failure to contact or attend the SGBV centre are conjectural. We think it likely, however, that issues of (i) transport (most of the women, 68%, lived in rural areas at some distance from the town where the SGBV Shelter and services were located, and were of low economic status) and (ii) privacy, may have contributed significantly (shame, stigma, fear of family break up and lack of agency associated with these experiences cannot be overestimated and other studies have also described fears of reprisal or of not being believed or supported 6, 15, 16).

If we are correct in our assumptions, then introducing not just screening but also support services more locally in rural communities might offer solutions to these diffaculties and result in more victims of SGBV receiving services.

There is no definite consensus internationally on whether there is a measurable improvement in health outcomes as a result of screening for SGBV 25. Some authors, with expertise in the psychological consequences of SGBV experiences, propose that there is qualitative evidence of the value of direct questioning with empathy, in treatment of the psychological aspects of women who have suffered in this way 25, 26. There is also the less direct but wider social value of regular SGBV screening in these and other health-care settings: over time, it would raise awareness among women of their rights under the law, it would contribute to education of the wider communities and to reducing stigma among victims and their families, removing some of the obstacles to help-seeking behaviours 27.

The difficulty of proving ultimate benefit, the uncertainty over whether the problem is sufficiently prevalent to warrant screening, and the emergence of both positive and negative consequences of screening 28 all contribute to this lack of consensus, though some of these specific issues may apply more particularly in high-income settings. Establishing screening tools that are both appropriate and feasible is also problematic. There are many screening tools developed particularly for Intimate Partner Violence (IPV), used in higher income countries, which have been subject to systematic reviews 29-31. The HITS (Hurts, Insults, Threatens and Screams) scale was considered the best short screening tool for health-care settings by Feder et al 32. However, the current context of a low-income country, several years post-conflict and with strong cultural elements of gender inequality, mandated a model better adapted to these conditions and a decision was made to use a tool designed for use in refugee screening at a Ugandan Refugee Camp 7 as likely to approximate more closely to these.

Our study, training staff so that a screening protocol could be piggybacked onto established Reproductive Health Service clinics and combining screening with a pathway to free care and treatment, offers a potential way forward in this difficult but extremely serious area.

### Limitations of the study

The study primarily assessed the feasibility of this screening project and its value in discovering undetected cases of SGBV by utilising Reproductive health workers. We do not claim to uncover all cases.

By its nature (using reproductive health services) our study did not include screening for male victims of SGBV – although the majority of SGBV is against women, in some estimate's male sufferers comprise up to 14% 8, 14, 21, 33, 34. Males questioned in other studies have expressed feelings of being discriminated against by their grievances being neglected by concentrating on female sufferers and the free services offered to them 8, 14, 21, 27, 33, 34. Nor would our study have revealed cases where victims did not attend specifically reproductive health services.

Women who had experienced SGBV were not asked if they had already received any help for their experiences, and an assumption was made that they had not, which may not have been the case in all the women screened. This may have been an additional reason for the relatively small number who chose referral to the SURGE services. The passage of time since the incident may also have contributed to the relatively high numbers screening positive but not seeking help.

## Conclusions

We have shown that an investigative screening project using services already available locally but with some additional training, and taking advantage of existing expertise and experience, is an effective model for detecting undisclosed cases of SGBV.

This approach could be used for planning future strategy concerning the detection and management of SGBV. Our study could lay the groundwork for the expansion and development of services to rural communities, by a community-based model, using the same format as described in this screening project and based in Health Centres.

We postulate that this method of screening for SGBV could be incorporated into health service policy and could inform provision of services. Over time, the provision of such services as an accepted part of community health may assist women to come forward more willingly and reduce the stigma of so doing within their communities. It could also help to drive education and attitudinal change so that societal tolerance of SGBV is reduced.
